# The economic burden of deliberate self-poisoning: insight from a tertiary hospital in the Free State Province, South Africa

**DOI:** 10.11604/pamj.2020.36.35.22346

**Published:** 2020-05-26

**Authors:** Matthew Abiodun Benedict, Nathaniel Mofolo, Anthonio Oladele Adefuye

**Affiliations:** 1 Department of Family Medicine, Faculty of Health Sciences, University of the Free State, Bloemfontein, South Africa; 2 School of Clinical Medicine, Faculty of Health Sciences, University of the Free State, Bloemfontein, South Africa; 3 Division of Health Sciences Education, Office of the Dean, Faculty of Health Sciences, University of the Free State, Bloemfontein, South Africa

**Keywords:** Deliberate self-poisoning, Free State Department of Health, economic burden

## Abstract

Suicide rate in South Africa is contentiously rated among the top ten highest in the world. Deliberate self-poisoning (DSP) remains one of the common methods for suicide. The management of DSP often impose a significant economic burden on health services with a growing loss of resources. However, studies on the financial implications associated with the management of DSP cases in South Africa are scarce and no known study has investigated the financial implication of managing DSP in a resource strained health system as obtained in the Free State Department of Health (FSDoH). This present study investigated the financial implication of managing DSP in a state regional hospital in the Free State province and proffer efficient ways of utilizing limited available resources in DSP management. This was a descriptive, retrospective cross-sectional study in which clinical records of 212 DSP cases which presented during an 18-month period at the emergency department of a state regional hospital were reviewed. The incidence of DSP was higher among individuals who are females (66% females vs 34% males), unemployed (65.6%) in the age group 20-29 years (44.8%). DSP management cost an average of R50, 000 per month. Wasteful expenditures such as blanket requests for laboratory investigation accounted for 19% of the cost. These findings agree with prior studies that have reported that managing DSP could pose a huge direct financial burden on hospital expenditure and health service delivery. If future cost containment and quality of care are to be achieved in the Free State province, efforts must be made by healthcare personnel to combat wasteful and unnecessary expenditure during patient management. We hope that recommendations proffered by this current study will alleviate the financial burden of DSP management in the province.

## Introduction

Deliberate self-poisoning (DSP), a common method of suicide, is the intentional ingestion of substances never intended for human consumption or of more than a prescribed amount of medicinal substances, whether or not there is evidence that the act was intended to result in death [1]. The global incidence of DSP has been estimated to be between 0.4% to 10% [2], affecting more females than males [3-5] and occurring in any person in the age range 20-30 years [6, 7]. Similarly, in South Africa, between 9.5% and 11% of all non-natural deaths are suicide related [8], and intentional self-harm ranked 6^th^ amongst the causes of non-natural deaths thus, accounting for 0.8% of non-natural deaths and 0.1% of all causes of death in the year 2016 [9]. The management of DSP often impose a significant economic burden on health services with a growing loss of resources [10]. The direct cost of managing DSP relates to services rendered by numerous departments that include the police force, emergency departments, acute medical units, critical care facilities, psychiatry and psychology services as well as social services and primary care [11-13], while the indirect cost encompass losses in productive activity [14]. In a study conducted to determine the hospital cost of managing DSP in Turkey, Serinken *et al*. (2008) reports that the mean cost of managing a patient with DSP was $144.06 ± 90.83 [14]. Similarly, in the United Kingdom, DSP accounts for 170 000 general hospital attendances each year with an estimated direct hospital cost of £56 million [10]. Over the past decade, the Free State Department of Health (FSDoH) has been experiencing an on-going financial crisis mainly due to poor financial management systems, insufficient health system financing, increasing costs, financial unsustainability and lack of financial autonomy [15]. This translated to a fragmented healthcare system, increased staff shortages and poor service delivery [15-17]. In South Africa, studies on the financial implications associated with the management of DSP cases are scarce [18], and no known study has investigated the financial implication of managing DSP in a resource strained health system as obtained in the FSDoH. In the present study, we investigated the financial implication of managing DSP in a regional hospital in the Free State province and proffer efficient ways of utilizing limited available resources in DSP management.

## Methods

This was a descriptive, retrospective cross-sectional study of the files of patients who presented with DSP at Pelonomi Regional Hospital Emergency Department between January 1^st^, 2010 and June 30^th^, 2011. Pelonomi Regional Hospital is a tertiary/teaching hospital situated in Bloemfontein, Free State Province. It serves as the referral centre for patients from neighbouring district and regional hospitals. Its emergency department provides initial/emergency treatment and resuscitation to medical and surgical emergency cases. All DSP patients who presented for treatment during the 18-month study period were identified from the emergency department (ED) patient register. The clinical files of these patients were retrieved and reviewed for relevant information that were recorded on a data collection tool, designed by one of the authors. Information retrieved include, demography (age, gender and employment status) and laboratory investigations conducted such as full blood count (FBC), urea and electrolyte (U&E), liver function tests (LFT) and toxicology reports. In addition, information regarding patients´ ward admission as well as referral for psychological assessment were also retrieved. Costs of services were calculated using the National Department of Health approved uniform patient fee schedule [19], National Health Laboratory Service state price list [20] and Universitas Hospital pharmacology laboratory price list. Data were entered into an Excel spreadsheet (Microsoft Corp, Redmond, WA, USA) and analysed using the Statistical Package for the Social Sciences (SPSS, version 22; IBM Corp, Armonk, NY, USA). Results were summarised by descriptive statistics for continuous data and frequencies and percentages for categorical data.

**Ethical consideration:** the Ethics Committee of the Faculty of Health Sciences, University of the Witwatersrand (M120427) approved the study. The Chief Executive Officer of Pelonomi Regional Hospital gave permission to conduct the study. The study was anonymous and data were handled confidentially.

## Results

Two hundred and sixty cases of DSP presented at Pelonomi regional hospital emergency department during the 18-month period (1^st^ January 2010 to 30^th^ June 2011) accounting for 0.8% of all emergency department visits. However, only 212 (81.5%) of these cases were suitable for analysis and inclusion in this study.

**Demography:** the majority (66.0%, n = 140) of the patients were female while male made up 34.0% (n = 72). Highest incidence was found in the age group 20-29 years (44.8%, n = 94) and amongst unemployed individuals (n = 139, 65.6%). Students/scholars made up 19.3% (n = 41) while employed individuals made up 15.1% (n = 32) of the cases recorded during the study period (Table 1).

**Table 1:** Demographic pattern of DSP cases at Pelonomi regional hospital

	Frequency	
Variable	n	(%)
**Gender** (n = 212)		
Male	72	34.0
Female	140	66.0
**Age group in years** (n = 210)		
< 16	6	2.9
16-19	38	18.1
20-29	94	44.8
30-39	51	24.3
≥ 40	21	10.0
**Employment** (n = 212)		
Employed	32	15.1
Unemployed	139	65.6
Students/scholars	41	19.3

**Mode of transportation:** one hundred and forty-four (67.9%) patients were conveyed to the emergency department with the Emergency Medical Services (EMS) ambulance.

**Laboratory investigations:** as regards laboratory investigations, LFT, U&E and FBC were carried out on 131 (61.7%), 138 (65.1%) and 195 (92.0%) patients, respectively (N = 212). Abnormal LFT and U&E results were found in only 13.0% (n = 17) and 8.0% (n = 11) of cases, respectively. Of the 108 patients on whom toxicology screen was done, only 6.5% (n = 7) had a positive screen in for the primary agent/drug.

**Patient outcome:** of the 204 patients who had this information recorded, the majority, that is 62.3% (n = 127) were discharged from the emergency department. A third of the patients (36.8%, n = 75) were admitted to the different wards: medical ward 31.9% (n = 65), gynaecology ward 1.5% (n = 3), surgical ward 1.0% (n = 2), and intensive care unit (ICU) 2.5% (n = 5).

**Referrals:** only 81.4% (n = 166) of the patients were referred to a psychologist for assessment on an outpatient basis (N = 204).

**Cost estimates:** according to the 2017 National Department of Health approved uniform patient fee schedule, the full (unsubsidised) fee for emergency department consultation at a provincial/regional hospital is R480 per visit [19]. Daily cost of admission into the ward and ICU are R1, 221 and R7, 712, respectively. Psychologist outpatient consultation fee is R207 per patient. Toxicology screen cost about R1,000 per patient and the cost of running LFT, U&E and FBC on each patient is R445 (i.e. R115, R272 and R58, respectively) [20]. Transportation of each patient by an intermediate life support paramedic, using the state ambulance, costs R1,558 per case. The estimated cost for the management of each DSP case ranges from R3,690 (for a non-complicated case discharged from ED) to R42,250 (for a complicated case admitted to ICU for 5 days). The total estimated cost for the 212 cases was R895 026 (Table 2).

**Table 2:** Breakdown of accrued cost for the management of DSP cases at Pelonomi regional hospital

Hospital ward admission/treatment					
Unit	Length of stay	Number of patients	Basis	Unit fee (R)	Total (R)
ED	Single visit	212	Per visit	480	101 760
Ward	2 days	70	Per day	1 221	170 940
ICU	5 days	5	Per day	7 712	192 800
Psychology	Single visit	166	Per visit	207	34 362
Ambulance	-	144	Per ride	1 558	224 352
Sub-total					724 214
**Laboratory tests (NHLS and Pharmacology)**					
**Test**		**Number of patients**	**Basis**	**Unit fee (R)**	**Total (R)**
U&E		138	Per test	115	15 870
LFT		131	Per test	272	35 632
FBC		195	Per test	58	11 310
Toxicology screen		108	Per test	1 000	108 000
Sub-total					170 812
**Grand total**					**895 026**

ED: emergency department; FBC: full blood count; ICU: intensive care unit; LFT: liver function test; NHLS: National Health Laboratory Service; U&E: urea and electrolytes

## Discussion

Deliberate self-poisoning (DSP) is considered a major public health problem worldwide [21-23], and it is one of the most common reasons for visiting an emergency department (ED) [2]. Similarly, admissions for DSP are a common occurrence at many ED in South Africa [24]; however, there are limited information on profile of patients managed for DSP at district and regional hospitals in the Free State province and no known study has been conducted on the financial implication of managing DSP in the province. Our findings that showed a higher incidence of DSP in females (66% females vs 34% males) corroborates similar findings by Hoving *et al.* (2018) [25]. The peak age of DSP occurrence (20-29 years) presented herein is in accordance with similar findings by Ani *et al*. (2017) [24]. Unemployment status has been reported to be a major risk factor for suicide [26]. In a study carried out to investiagte suicide cases at the state mortuary in Bloemfontein, Free State South Africa, Stark *et al.* (2010) reports that 56.9% of the individuals who died by suicide were unemployed [27]. Similarly, findings presenetd by this study reveal that the majority (65.6%) of patient admitted for DSP during the study period were unemployed. Thus, suggesting that unemployment is a risk factor for DSP in the Free State Province. More so, the FSDoH recorded a youth (15-34 years) unemployment rate of 37.2% for the province in the year 2018/2019 [28]. It has been reported that the management of DSP can pose both direct and indirect financial burden on health services and health budget [10, 14]. Findings by this present study reveal that an average of R50,000 per month was spent on managing DSP at Pelonomi hospital (Table 2). Data presented by this study shows that approximately one fifth (19%) of the total expenditure on DSP cases accrued to laboratory tests (Table 2) and >80% of these tests were normal. If uncortailed, wasteful expenditure such as this will have a huge negative impact on the already financially constrained health sector in the province, consequently worsening the already poor healthcare service delivery. The present stituation warrants that all healthcare personnel should judiciously utilize the limited available resources during service delivery.

## Conclusion

Our findings are consistent with previous studies that have described the demographic profile of patient that present at ED with DSP. The results of the current study agrees with prior studies that have reported that managing DSP could pose a huge direct financial burden on hospital expenditure and health service delivery. If future cost containment and quality of care are to be achieved in the Free State province, efforts must be made by healthcare personnel to combat wasteful and uncessary expenditure during patient management. Recommendations: based on the findings of this study, the researchers propose the following recommendations to alleviate the financial burden of DSP management at Pelonomi hospital: 1) develop a framework for saving cost: a cost saving framework that is complimentary to the current DSP management approach should be developed. 2) Avoid blanket request for laboratory test: laboratory investigations for DSP management should be individualised, based on the clinical presentation and only relevant tests should be requested. 3) Develop a protocol for managing common cases: it has been reported that subatance of poisoning vary according to geographical location and season [29]. Thorough regular audit processes should be carried out by every health facility that manages DSP cases to determine the top agents/substance ingested. 4) Lay emphasis on prevention rather than cure: a british study showed that about 45% of adults who committed suicide visited their primary care provider shortly before suicide [30]. Screening tools such as the Beck Scale for Suicide Ideation (BSS) might be useful for early identification of at risk individuals, who could subsequently be referred for psychological intervention. 5) Limit factors that increases length of hospital stay: according to protocol, patients admitted on account of DSP have to consult with social worker or psychologist prior to discharge; a delay in rendering this service will ultimately result in additional cost. Necessary review/assessment of these patients should be done timeously so that decision can be made swiftly.

### What is known about this topic

- DSP is a common method of suicide and it is a major public health problem worldwide;
- The management of DSP often impose a significant economic burden on health services;
- Admissions for DSP are a common occurrence at many ED in South Africa.

### What this study adds

- This study confirmed that the demographic profile of patient managed for DSP in the Free State Province is similar to findings in the literature;
- This study contextualised the economic burden of managing DSP in a tertiary hospital in the Free State Province;
- The present study provides implementable recommendations on how to alleviate the financial burden of DSP management in a financially constrained health system.

## Competing interests

The authors declare no competing interests.
